# Microstructure, Mechanical, and Corrosion Behavior of Al_2_O_3_ Reinforced Mg2Zn Matrix Magnesium Composites

**DOI:** 10.3390/ma14174819

**Published:** 2021-08-25

**Authors:** Ali Ercetin, Danil Yurievich Pimenov

**Affiliations:** 1 Department of Mechanical Engineering, Faculty of Engineering and Architecture, Bingol University, Bingol 12000, Turkey; 2 Department of Automated Mechanical Engineering, South Ural State University, Lenin Prosp. 76, 454080 Chelyabinsk, Russia

**Keywords:** metal matrix composites (MMCs), magnesium matrix composites (MgMCs), microstructure, mechanical properties, corrosion, powder metallurgy, hot pressing method

## Abstract

Powder metallurgy (PM) method is one of the most effective methods for the production of composite materials. However, there are obstacles that limit the production of magnesium matrix composites (MgMCs), which are in the category of biodegradable materials, by this method. During the weighing and mixing stages, risky situations can arise, such as the exposure of Mg powders to oxidation. Once this risk is eliminated, new MgMCs can be produced. In this study, a paraffin coating technique was applied to Mg powders and new MgMCs with superior mechanical and corrosion properties were produced using the hot pressing technique. The content of the composites consist of an Mg2Zn matrix alloy and Al_2_O_3_ particle reinforcements. After the debinding stage at 300 °C, the sintering process was carried out at 625 °C under 50 MPa pressure for 60 min. Before and after the immersion process in Hank’s solution, the surface morphology of the composite specimens was examined by scanning electron microscopy (SEM) and energy dispersive spectroscopy (EDS) analysis. With the hot pressing technique, composite specimens with a very dense and homogeneous microstructure were obtained. While Al_2_O_3_ reinforcement improved the mechanical properties, it was effective in changing the corrosion properties up to a certain extent (2 wt.% Al_2_O_3_). The highest tensile strength value of approximately 191 MPa from the specimen with 8 wt.% Al_2_O_3_. The lowest weight loss and corrosion rate were obtained from the specimen containing 2 wt.% Al_2_O_3_ at approximately 9% and 2.5 mm/year, respectively. While the Mg(OH)_2_ structure in the microstructure formed a temporary film layer, the apatite structures containing Ca, P, and O exhibited a permanent behavior on the surface, and significantly improved the corrosion resistance.

## 1. Introduction

State-of-the-art biomaterials help a living structure within the body to accomplish, enhance, or substitute its natural function. They can form all or part of the living structure, as needed. The most important feature of a biomaterial is that it is biocompatible. In other words, it is non-toxic [1] and does not harm living tissues in the area where it is used [2]. Ti6Al4V, CoCr, stainless steel, platinum, and the like, which are permanent biomaterials [3], pose minimal danger in the body as they dissolve in the body at very low level [4]. However, when these materials are applied temporarily, such as implants [5], they must be removed from the body by a second surgical operation when the diseased area heals [6]. In addition, these biomaterials traditionally used metallic materials, which often exhibit unsatisfactory results such as stress protection, metal ion release, or allergic reaction. When the implant has a toxic effect on the body, secondary surgical operation(s) is usually unavoidable [7]. Stent applications used in cardiovascular surgery can condemn the patient to the use of blood thinners and similar drugs for life. Therefore, the use of biodegradable materials is becoming more and more important for temporary and short-term treatments. Magnesium-based biomaterials are candidates for use as a new generation of biodegradable metals [7].

Although Mg-based biomaterials have biodegradable properties in in-body applications [8,9], they also contain some basic elements (Mg, Zn, Sn, Ca, etc.) needed by the body [10,11]. Moreover, the density and elasticity modulus values of pure Mg exhibit properties quite close to those of human bones [12]. However, the low corrosion resistance of pure Mg has shown that AZ61 [13,14], MgSnZn [13,15] new alloys and MgZnCa-(nHA reinforced) [16], Mg-(HA reinforced) [17], AZ31-(CNT reinforced) [18], and MgZrZn-(graphene nanoplatelet (GNP) reinforced) [19] biocomposites should be developed.

One of the most important factors affecting the corrosion resistance of Mg alloys is the intermetallic phases formed at the grain boundaries [15,20,21,22]. Depending on the increasing amount of intermetallic phase, different polarizations (anode and cathode) occur between the grain boundaries and the grain interiors. As a result of this situation, galvanic corrosion occurs [15,23,24]. Zn is one of the important alloying elements that increase the mechanical properties and corrosion resistance of Mg, and has biocompatible properties [15,25]. According to the Mg-Zn binary phase diagram [9,26], the solubility of the alloying element Zn in Mg is 6.2 wt.% at 325 °C [27], and at room temperature it is around 2 wt.%. During the rapid cooling to room temperature of the Mg alloy containing more than 2 wt.% Zn, intermetallic phase formation may start at the grain boundaries [26]. Therefore, it is expected that the rate of 2 wt.% should not be exceeded.

New Mg alloys with improved mechanical and corrosion properties have been produced by adding Al [28], Zn [29], Ca [30], Sn, and Mn [31] and various alloying elements to Mg [32]. However, for some applications, Mg alloys may still be insufficient. Therefore, studies on the production of Mg matrix composites have also gained momentum [10,30,33]. In the production of Mg matrix composites, two important issues should be considered in the selection of the reinforcement to be added. First, it must be biocompatible and have good corrosion resistance. Secondly, the mechanical strength of the reinforcement should be better than the matrix of the composite [13,30,34,35,36]. It is possible to say that ceramic reinforcement elements such as Al_2_O_3_, SiC, and HA (hydroxyapatite) can meet these requirements [9,13,37,38].

In some studies on MgMC, while the effect of the reinforcement addition on corrosion properties was investigated, Zn was generally used in the content of the matrix alloy [30,39,40]. Since the solubility of Zn in Mg at room temperature is maximum 2 wt.%, Mg2Zn alloy was chosen as the matrix [39,41]. This is the reason why Mg2Zn alloy was chosen as the matrix in the current study. The effect of Al_2_O_3_ reinforcement particles added at different proportions on hardness and corrosion properties was investigated. The paraffin coating technique facilitated the production of Mg-based materials with powder metallurgy. The hot pressing method (one of the PM methods) has been used as the production method to ensure the production of composite specimens with high relative densities, and to ensure a homogeneous distribution of reinforcement particles in the microstructure.

## 2. Materials and Methods

### 2.1. Mixing Process, Production, and Microstructural Investigation

The properties of the Mg and Zn powders used in the formation of the matrix alloy, and the chemical compositions of the Mg2Zn alloy, are given in Table 1. The properties of Al_2_O_3_ powder used as reinforcement additions in different proportions and the chemical compositions of Mg2Zn-x Al_2_O_3_ composites are given in Table 2. The powders used are of micron size and have high purity.

**Figure 1:**
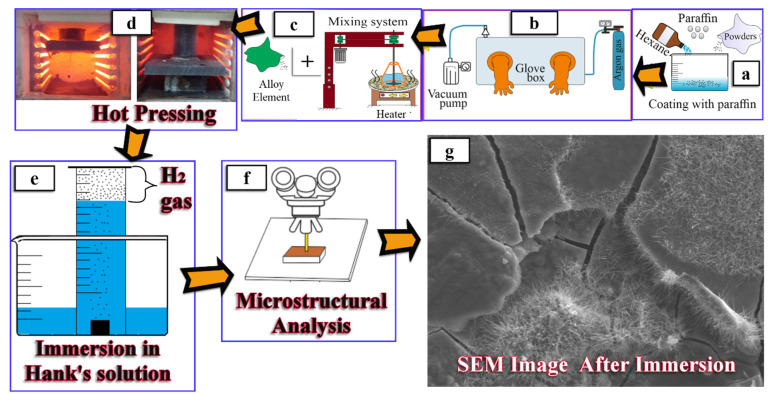
Graphical presentation of (**a**) paraffin coating of powders, (**b**) glove box system, (**c**) adding alloying elements, (**d**) hot pressing, (**e**) measuring evolved H_2_ gas, (**f**) microstructural analysis, (**g**) a SEM image after immersion process.

During the weighing and mixing process of the powders, there is a risk of oxidation due to the contact of Mg powders with air. A paraffin coating technique was applied to the Mg powders to prevent this risk. Mg powders were supplied by the Alfa Aesar Company in a 500 g vacuum package. After the gross weight of the package was weighed, it was opened in a glove box and the Mg powders were poured into a beaker containing hexane. In determining the net weight of the Mg powders, the difference between the (gross) weight and the empty weight (tare) of the package was calculated. Precision scales (Precisia brand, Dietikon, Switzerland) with an accuracy of 10^−4^ g were used in the weighing processes. Before opening the package in the glove box, the cabin was filled with high purity argon gas and then vacuumed. The volume of the Mg powders was calculated by the ratio of its net weight to its density, and 20% by volume of paraffin was added to the hexane + Mg powders in the beaker (Figure 1a,b). The beaker containing the mixture was placed on a heater to completely dissolve the paraffin in hexane and was heated up to 70 °C (Figure 1c). While the beaker was on the heater, a propeller mixer was immersed into the mixture. Stirring was continued at 180 rpm for 60 min until all the hexane had evaporated. The same mixing procedures were repeated for the preparation of the mixtures specified in Table 1 and Table 2. Important studies have been carried out to show that the mixing speed, time, and sintering temperature affect the microstructure, mechanical, and corrosion properties [42,43,44]. Therefore, in this study, mixing speed, time, and sintering parameters have been examined in previous studies [15,32,45], and the optimum production parameters were applied.

Specimens of each mixture were produced in a hot press bench using a graphite mold system of 30 mm length, 10 mm width, and 3.5 mm dimensions. The hot pressing system (Hidro Metal brand, Konya, Turkey) used in the study has a maximum temperature of 1000 °C and a capacity of 400 kN (Figure 1d). After 30 min of debinding at 300 °C, sintering processes were carried out using high purity argon gas at 625 °C under 50 MPa pressure for 60 min. Sanding, polishing, and etching processes were applied to the produced specimens, respectively. The solution of 5 vol.% nitric acid and 95 vol.% absolute ethyl alcohol, which is frequently used in the etching process of Mg based materials [46,47], was also used in the present study. SEM and EDS-mapping analyses were applied for the microstructure and phase examinations of the specimens. ImageJ software was used to measure average grain sizes of specimens. XRD analysis was carried out to determine phase formations of specimens before and after immersion tests. Measured densities of specimens were determined by following ASTM B962-14 [32,48]. According to this standard, also known as the Archimedes principle, the specimens were first weighed in air and then weighed in distilled water [32,48]. The theoretical density was calculated according to Equation (1). The measured density/theoretical density ratio was used in the calculation of (%) relative density values.

(1) $$\mathrm{Theoretical}\;\mathrm{density}\;(g/{\mathrm{cm}}^{3})\;=\;\frac{Total\;mass}{Total\;volume}\;=\;\frac{\left(Mg\;mass\right)\;+\;\left(Zn\;mass\right)\;+\;\left({Al}_{2}O_{3}\;mass\right)}{\left[\frac{Mg\;mass}{Mg\;density}\right]\;+\;\left[\frac{Zn\;mass}{Zn\;density}\right]\;+\;\left[\frac{{Al}_{2}O_{3}\;mass}{{Al}_{2}O_{3}\;density}\right]}$$

### 2.2. Mechanical and Corrosion Test

Hardness tests were applied for 15 s with 300 g load, to determine the mechanical properties of the specimens. A micro Vickers hardness tester (AOB Labtt brand, İstanbul, Turkey) was used to obtain the hardness values. The average of the measurements taken from five different regions of each specimen was accepted as the hardness value. A shimadzu tensile test device with a capacity of 10 kN was used for tensile tests in air at room temperature. Pull rate was chosen as 0.5 mm/min. Rectangular prism-shaped specimens (30 × 10 × 3 mm) were cut with wire. The produced specimens were formed into tensile specimens according to the MPIF-10 standard.

Corrosion tests were carried out according to standards reported in the literature [15,28,49]. Weight loss (%) and evolved H_2_ gas measurements were considered in determining the corrosion behavior of Mg2Zn-xAl_2_O_3_ specimens. In the tests, specimens were subjected to sanding and polishing in 10 × 10 × 3 mm (length × width × height) dimensions and Hank’s solution [15] were used as the corrosion fluid. The ratio of corrosion liquid to total surface area of specimen was applied as 20:1 (mL/cm^2^). When the specimens were placed in the corrosion liquid in the beaker, a graduated measure, where the evolved H_2_ gas could accumulate, was placed on the specimen. The specified system was used for 10 days in an incubator at 37 °C constant temperature (Figure 1e). The corrosion fluid was renewed every 12 h, and the H_2_ gas accumulated in the cylinder was measured. When the specimens were taken from the corrosion liquid, they were firstly cleaned with pure ethyl alcohol and then placed in a desiccator to dry. After the drying process was applied to the specimens, their weight was measured. The specimen weight loss (%) was calculated according to Equation (2). After the 10-day corrosion process was completed, the specimen’s surface structure was examined with SEM and EDS (Figure 1f,g).

(2) $$\mathrm{Weight}\;\mathrm{loss}\;\mathrm{accuracy}\;(\%)\;=\;\frac{\left(Last\;weight\;-\;First\;weight\right)\;of\;specimen}{First\;weight\;of\;specimen}\;\times \;100$$

## 3. Results and Discussions

**Figure 2:**
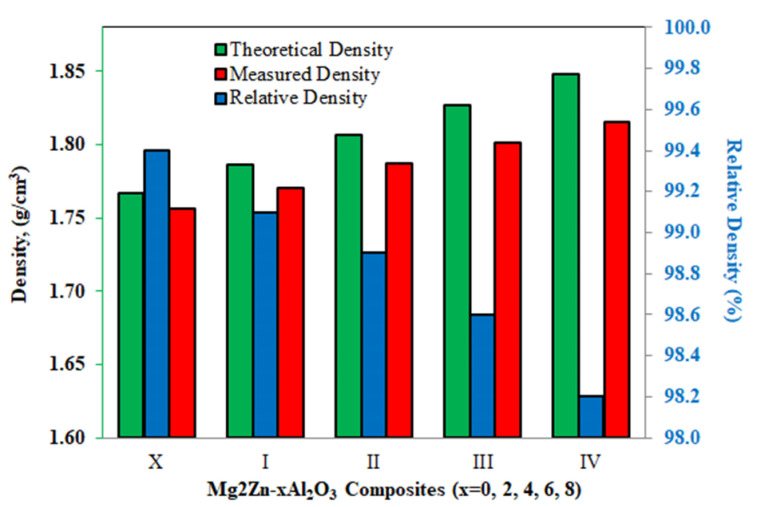
Theoretical, measured, and relative density values of Mg2Zn-x Al_2_O_3_ composite specimens.

The changes of theoretical density, measured density, and relative density data, depending on the increasing reinforcement ratio, are given in Figure 2. The theoretical and measured density values increase with increasing reinforcement ratio. However, the relative density values of specimens with high reinforcement additions are lower than the other specimens. It has been frequently stated in the literature that the sintering process is successfully performed when the relative density values of the materials produced by powder metallurgy method are above 95% [17,43]. In the present study, even the lowest relative density value was higher than 98%, indicating that the Mg2Zn-xAl_2_O_3_ composite specimens were successfully produced by the hot pressing method.

### 3.1. SEM and EDS Analysis of Specimens before Immersion

**Figure 3:**
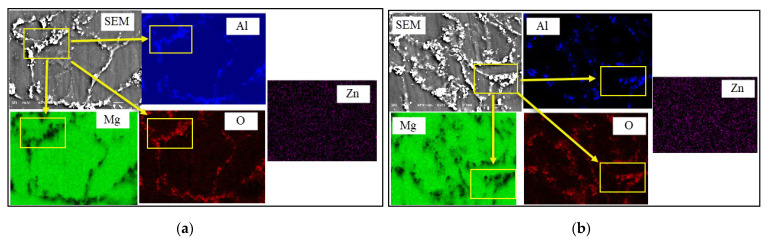
SEM/EDS mapping analysis before immersion (**a**) Mg2Zn-2 wt.% Al_2_O_3_ magnesium composite, (**b**) Mg2Zn-8 wt.% Al_2_O_3_ magnesium composite.

The EDS-mapping analysis images of the specimens containing 2 wt.% Al_2_O_3_ and 8 wt.% Al_2_O_3_ are given in Figure 3a,b, respectively. The regions (inside matrix) which have a rich Mg content are shown in a green color. The Mg content is more intense in the grains. The Zn content can be seen in every region (pink color). If the mapped distributions of Mg, Al, and O elements are examined together, the Mg content in the grain boundary regions is almost nonexistent (black color), while the contents of Al (blue color) and O (red color) are much richer in the same regions. According to this result, it is understood that the white structures in the grain boundaries belong to the Al_2_O_3_ reinforcement particles.

**Figure 4:**
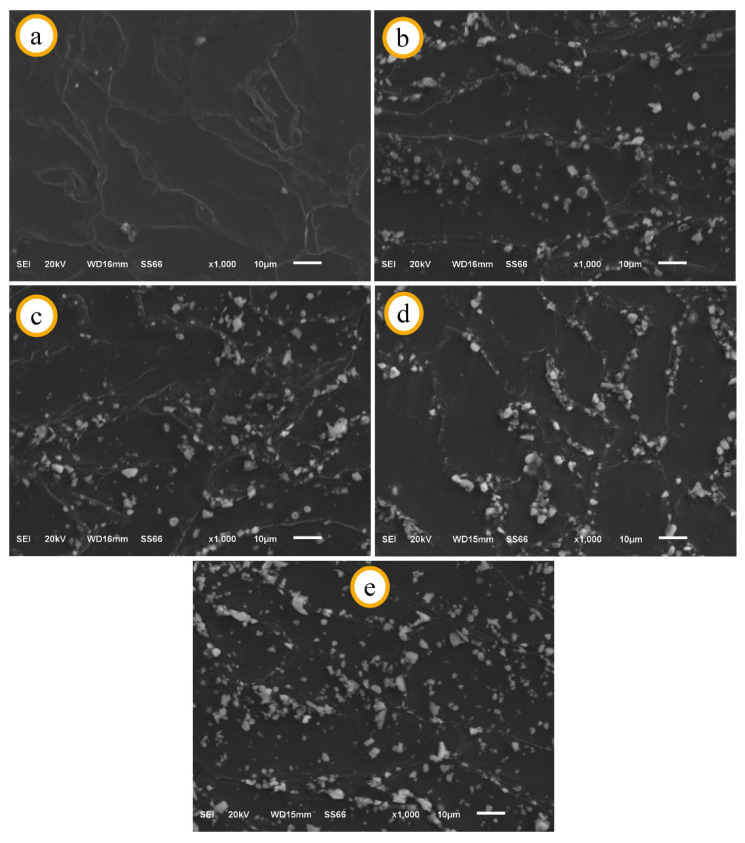
SEM images (at 1000× magnification) of Al_2_O_3_ reinforced Mg2Zn matrix magnesium composites before immersion: (**a**) X, (**b**) I, (**c**) II, (**d**) III, (**e**) IV.

SEM images of Mg2Zn-xAl_2_O_3_ composite specimens are given in Figure 4. Grains and grain boundaries can be clearly seen in all the sintered specimens. From the SEM images, it can be seen that all the specimens have a very dense microstructure. Additionally, no pore structures were found in the microstructure. The microstructural images also support the relative density graphs in Figure 2. Al_2_O_3_ particles are homogeneously distributed in the grain boundaries of the Al_2_O_3_ reinforced specimens. It was also observed that there is no clustering of reinforcing particles in any region. It has been determined that the production method (PM method) is considerably effective in obtaining a homogeneous microstructure, both in the present study and in other literature studies [10,47,50]. With the increasing rate of reinforcement, the amount of Al_2_O_3_ particles at the grain boundaries increases and the grain sizes are generally smaller. It is thought that specimens with higher content of Al_2_O_3_ have finer-grained microstructures, as the reinforcement particles act as barriers. Average grain sizes of the specimens X, I, II, III, and IV are 34.82 µm, 29.96 µm, 29.21 µm, 23.72 µm, and 18.75 µm, respectively. For similar studies reported in the literature, it was determined that grain growth was prevented due to the increasing rate of hard structures homogeneously distributed at the grain boundaries [47,51,52].

The SEM images also show that all specimens achieved a good degree of wettability. As a wettability criterion, there is no pore at the contact points of the reinforcement particles and the Mg matrix. The use of Mg alloy as a matrix is effective in the high wettability of the specimens. In a different study, Suresh et al. [53] found that Mg increases the wettability properties of composite materials.

**Figure 5:**
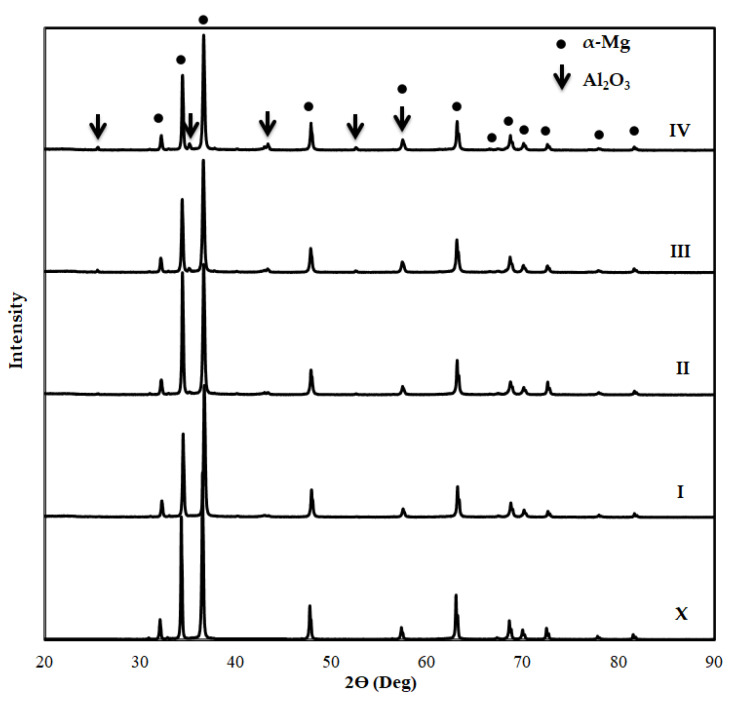
XRD patterns of Al_2_O_3_ reinforced Mg2Zn matrix magnesium composites before immersion.

After the Mg2Zn-xAl_2_O_3_ composite specimens were produced through hot pressing method, XRD analysis was applied to all specimens to determine phases in the microstructure. Figure 5 shows the XRD patterns of specimens before immersion test. First of all, when the XRD patterns of the Mg2Zn alloy without alumina reinforcement were examined, no oxygen-containing phase was found. While the peaks of the Mg phase were obtained in all specimens, the peaks of the Al_2_O_3_ phase could be seen more clearly with the increasing alumina reinforcement. The solubility of Zn alloying element in Mg at room temperature is about 2 wt.%. Therefore, the inability to obtain any peak of the Zn alloy element in the matrix in the XRD analysis was attributed to this situation. In similar literature studies [15,31,54], no peak could be detected in XRD analyses when the Zn content added to magnesium was less than 4 wt.%.

### 3.2. Corrosion Properties of Mg2Zn-xAl_2_O_3_ Composites

**Figure 6:**
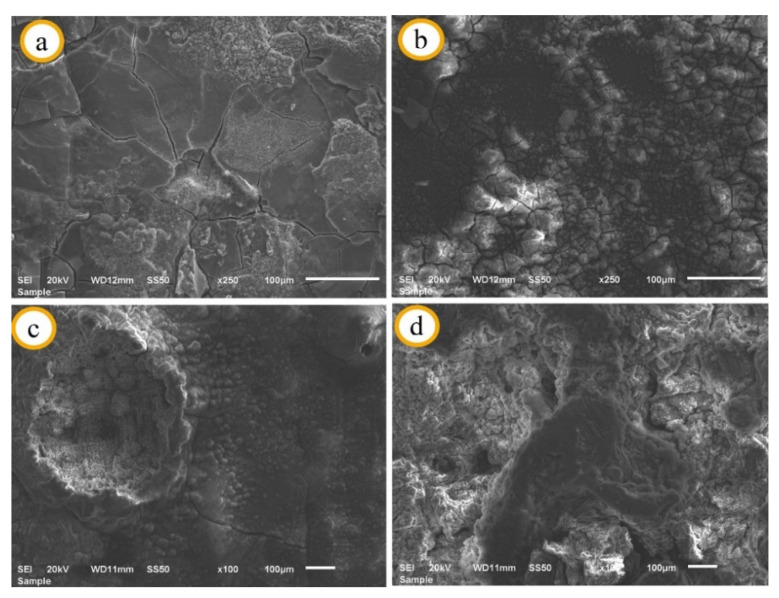
SEM images after immersion; (**a**) X, (**b**) I, (**c**) II, (**d**) III.

SEM images taken from the surfaces of the specimens (X, I, II, and III) after 10 days of immersion are given in Figure 6. The specimen (IV), containing 8 wt.% Al_2_O_3_, was exposed to degradation due to excessive corrosion at the end of the 10-day immersion period, and the SEM image of this specimen could not be taken. It can be seen that the surface structures of the specimens X and I are in good condition (Figure 6a,b), and large pitting corrosion has occurred on the surfaces of the specimens (II) and (III) (Figure 6c,d). The pits on the surface of specimen (III) are greater and larger than those on the surface of specimen (II). In the pre-corrosion SEM examination of the specimens, when the reinforcement addition was more than 2 wt.%, the amount of Al_2_O_3_ particles at the grain boundaries increased. It is thought that the grain interior (anode) and grain boundaries (cathode) behave as different poles during corrosion [23,24], and pits on the surface (Figure 6c,d) are formed as a result of galvanic corrosion. On the surface of the Mg2Zn matrix alloy (Figure 6a), it can be seen that different layer structures are formed in some regions, and the layer formed in certain regions is detached from the surface. Moreover, there are many and very long cracks on the surface. No pitting corrosion occurred on the surface of the specimen (I) with dense white structures being detected on the surface (Figure 6b).

During the corrosion of Mg in Hank’s solution, the reactions in Equations (3)–(6) occur [54,55,56]. According to Equation (5), the higher the formation of Mg(OH)_2_ corrosion product on the specimen surface, the higher the H_2_ gas volume evolved at the same rate.

Mg → Mg^2+^ + 2e^−^ (anodic reaction) (3)

2H_2_O + 2e^−^ → 2OH^−^ + H_2_ (cathodic reaction) (4)

Mg^2+^ + 2OH^−^ → Mg(OH)_2_ (product formation) (5)

Mg + 2H_2_O → Mg(OH)_2_ (corrosion product) + H_2_ (6)

**Figure 7:**
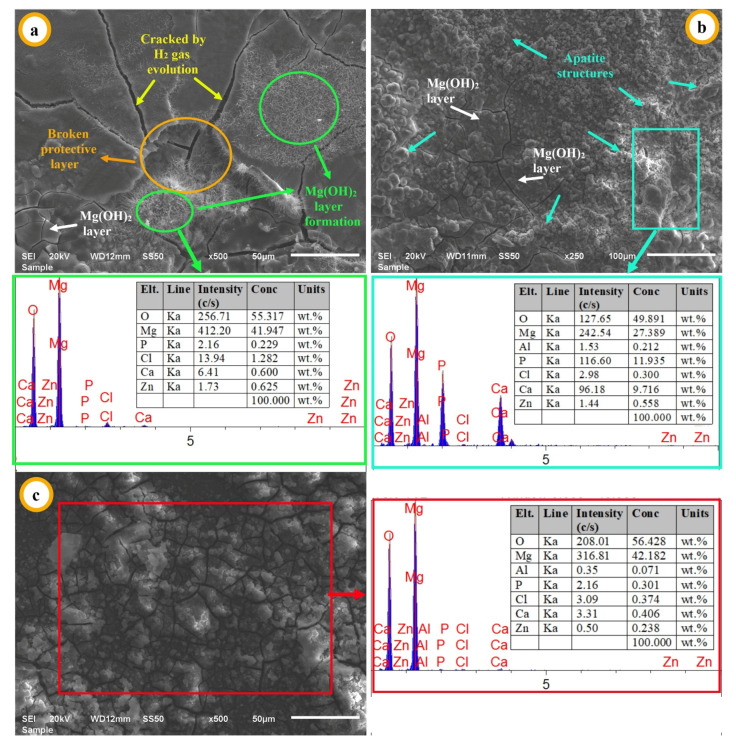
EDS analysis after immersion: (**a**) X, (**b**) I, (**c**) II.

EDS analysis, elemental peaks and contents of specimens (X, I, and II) are given in Figure 7. In Figure 7a, the structures indicated with green circles are very rich in Mg and O content. Therefore, it is thought that the Mg(OH)_2_ layer was newly formed in the specified green areas. The Mg(OH)_2_ film, which forms as layers on the surface after a certain period of time, is broken by the effect of the continuously evolved H_2_ gas. When the cracks where H_2_ gas is evolved are deeper and wider, the protective layer between the cracks can break off the surface (orange-colored circle). The surface under the separated layer comes into contact with the corrosion fluid and the corrosion reaction cycle continues. As the number of repetitions of this cycle increases, pitting corrosion occurs as seen in the SEM images in Figure 6c,d. When the temporary Mg(OH)_2_ film layer formed on the specimen surface remains for a longer period without leaving the surface, it is expected to react according to Equation (7) [15,55]. After this reaction, the reaction in Equation (8) is also expected to occur, and permanent and protective apatite structures are formed on the surface [10]. When the Figure 7b was examined, it was determined by EDS elemental analysis that the reactions stated in Equations (7) and (8) took place mostly on the surface of the specimen (I) containing 2 wt.% Al_2_O_3_. The region indicated on the SEM image with the turquoise-colored rectangle is very rich in terms of Mg, Ca, P, and O contents. It was determined that the layers indicated with a turquoise arrow and rectangle belong to the apatite structures. Apatite structures fill the cracks where H_2_ gas is evolved and serve as a kind of coating on the specimen surface. According to the findings reported in the literature [10,15], the apatite structures significantly improve the corrosion resistance. In the present study, it is thought that the corrosion properties of the specimen (I) with apatite structure are better. In Figure 7c, it is thought that the loose products were separated from the surface in a short time period and this period was completed before the Mg(OH)_2_ layer on the products could not transform into apatite structures. Detection of low content of Ca and P on the surface is associated with this situation according to EDS analysis of surface in Figure 7c.

Mg(OH)_2_ + 2Cl^−^ → MgCl_2_ + 2OH^−^ (7)

H_2_PO_4_^−^ + Ca^2+^ + Mg^2+^ + OH^−^ → Mg_x_Ca_y_(PO_4_)_z_ (insoluble products) (8)

**Figure 8:**
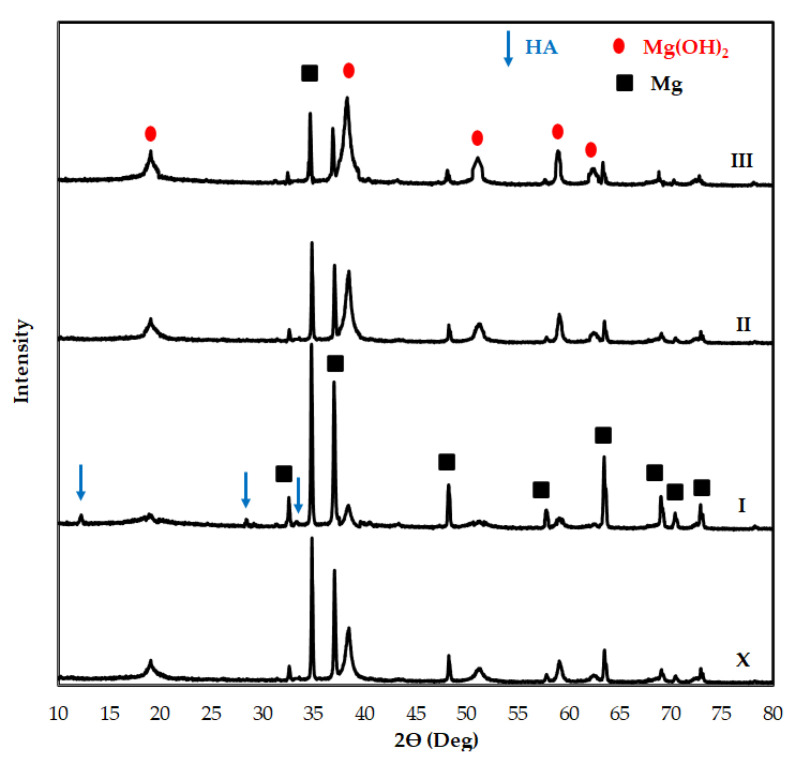
XRD patterns of Al_2_O_3_ reinforced Mg2Zn matrix magnesium composites after immersion.

Figure 8 shows the XRD analysis of Al2O3 reinforced Mg2Zn matrix magnesium composites after immersion. The peaks at 2θ = 19.16°, 38.30°, 51.05°, 58.94°, and 62.32° show the Mg(OH)_2_ phases. Mg peaks are obtained at 2θ = 32.58°, 34.72°, 37.02°, 48.26°, 57.90°, 63.48°, 68.98°, 70.36°, and 72.86°. The obtained findings are compatible with the literature studies [57,58]. The Mg(OH)_2_ phase ratio is high on specimen surfaces containing high Al_2_O_3_ reinforcement. On the other hand, the Mg phase ratio is at low levels. Mg phases decrease with the increase of Mg(OH)_2_ phases. This indicates that a large amount of Mg is converted to Mg(OH)_2_ formation on the surface. HA peaks can be clearly seen in the XRD patterns of specimen (I) at 2θ = 12.24°, 28.40°, and 33.38°. The fact that the HA (Ca_10_(PO_4_)_6_(OH)_2_) phases are more prominent in the specimen (I) is based on the corrosion inhibition property of the added Al_2_O_3_ reinforcement at low content (2 wt.%). It is known that the anode and cathode polarization increases with the increasing alumina reinforcement ratio and the desired resistance to galvanic corrosion cannot be demonstrated [57,59]. Therefore, in SEM images after immersion, pitting corrosion was observed in specimens containing high Al_2_O_3_. Pitting corrosion was not observed due to the formation of HA structure on the surface of the specimens containing 2 wt.% Al_2_O_3_.

It is well known that Al_2_O_3_ has a good corrosion resistance. While corrosion resistance is expected to increase with increasing Al_2_O_3_ reinforcement ratio, corrosion resistance decreased in specimens containing more than 2 wt.% Al_2_O_3_ reinforcement. This is attributed to the formation of sufficient time period for the Mg(OH)_2_ structures that are constantly present on the surface to transform into HA structures. As stated earlier in the SEM/EDS mapping analysis, Al_2_O_3_ particles are observed to be more dense at the grain boundaries with increasing Al_2_O_3_ reinforcement ratio. This increases the interface between matrix and reinforcement. It is reported that reduction of galvanic corrosion between grain boundary and matrix enhanced corrosion resistance [60]. Therefore, in the present study, high activity of the galvanic corrosion occurred in specimens containing high Al_2_O_3_ reinforcement. It is also thought that there is not enough activity of galvanic corrosion to reduce the time period required for the formation of apatite structures on the surface of the specimen containing 2 wt.% Al_2_O_3_. It is reported in a similar study [61] that the increase of reinforcement ratio in the magnesium matrix composites causes high activity of the galvanic corrosion.

**Figure 9:**
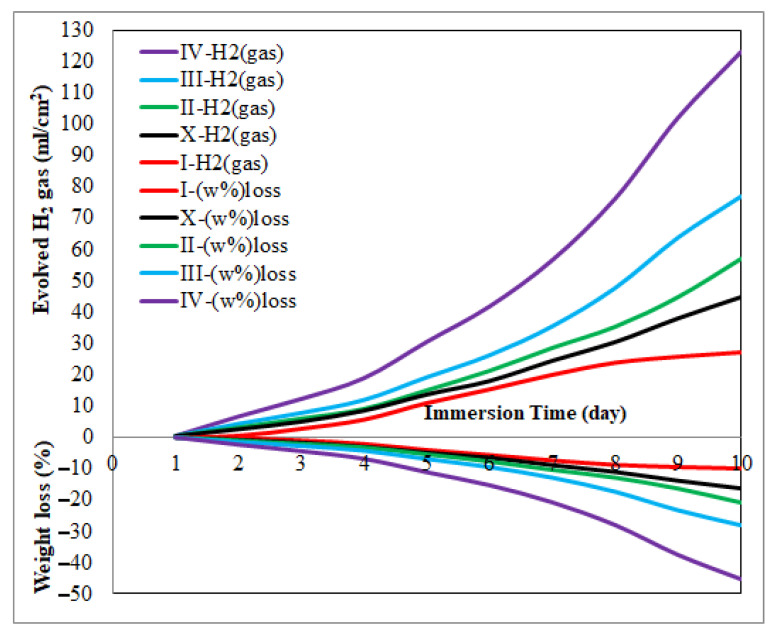
Weight loss (%) and evolved H_2_ gas (ml/cm^2^) after 10 days of immersion.

After the immersion process was applied for 10 days, the daily (%) weight loss of the specimens and the evolved H_2_ gas values are given in Figure 9. With the increase in the immersion time, both the weight loss and the amount of evolved H_2_ gas increase. While the corrosion rate increased continuously in all specimens, the corrosion rate of the 2 wt.% Al_2_O_3_ reinforced composite specimen stopped growing from the 7th day. The best corrosion properties were also obtained from this specimen. With the addition of more reinforcement than this ratio, the corrosion properties were negatively affected. The reason why this specimen exhibits better corrosion behavior compared to other specimens is the apatite structures whose presence on the surface was detected in SEM-EDS examinations. In specimens with high reinforcement ratios, it is thought that the dense reinforcement particles at grain boundaries trigger galvanic corrosion and negatively affect the corrosion resistance.

Another notable observation in Figure 9 is that a small amount of H_2_ gas is evolved from the specimen with low weight loss and the amount of evolved H_2_ gas increases as the weight loss rate increases. When Equation (4) and XRD analysis after immersion are examined together, the amount of evolved H_2_ gas is directly associated with the formation of Mg(OH)_2_ layer on the surface. The pitting corrosion causes on the surface during immersion means that the interface between the specimen and the corrosion liquid increases. This means more Mg(OH)_2_ formation and more evolved H_2_ gas. Therefore, the amount of evolved H_2_ gas also increases with the increase in weight loss ratio (Figure 9). In a study on corrosion of MgMC [17], HA in different proportions was added to the Mg3Zn matrix and the composite was subjected to an immersion process. The composite specimen with the addition of 5 wt.% HA, for which they achieved the best results, lost about 27% weight. In the present study, the lowest weight loss was obtained from the specimen containing 2 wt.% Al_2_O_3_ at approximately 9%. This value is 18% less than the result obtained by Dubey et al. In a different study, Jayalakshmi et al. [20] examined the corrosion properties of AM100 and ZC63 magnesium alloys by adding different amounts of saffil alumina short fiber reinforcement by volume. They obtained the best corrosion properties at approximately 7 mm/year, and approximately 9.5 mm/year from AM100 + 25 vol.% alumina and ZC63 + 25 vol.% alumina specimens, respectively. In the present study, a weight loss of 9% corresponds to approximately 2.5 mm/year when converted to the unit system mm/year. This value is 280% less than AM100 + 25 vol.% alumina and 380% less than ZC63 + 25 vol.% alumina. A comparative presentation of the corrosion properties obtained in present study with some literature studies is given in Table 3.

### 3.3. Mechanical Properties of Mg2Zn-xAl_2_O_3_ Composite Specimens

**Figure 10:**
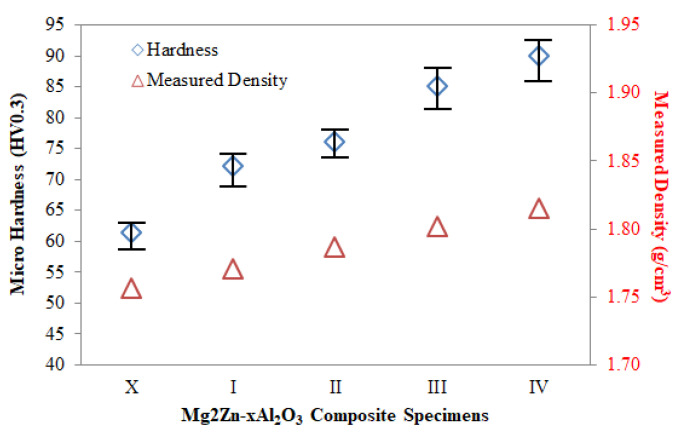
The micro hardness values of Mg2Zn-xAl_2_O_3_ composite specimens.

Figure 10 shows the hardness values of Mg2Zn-xAl_2_O_3_ composite specimens. When the graph is examined, it can be seen that particle reinforcement has a positive effect on the hardness properties. It is thought that the increase in hardness occurs due to two reasons. The first is that the particle reinforcements, which are harder than the grain interiors (Mg2Zn matrix), have a reticulated structure at the grain boundaries. The other is that finer-grained structures were obtained due to Al_2_O_3_ particles acting as barriers at grain boundaries. Kandemir et al. [33] used AZ91 magnesium alloy, which is widely used in the industry, as a matrix and added up to 0.5 wt.% GNP. The highest hardness value from their composites was 69 HV. In a study by Lotfpour et al. using the same matrix alloy [41], they added Cu in different proportions to the Mg2Zn matrix. They obtained a highest hardness value of approximately 78 HB from the material with 5 wt.% Cu added. This value corresponds to a hardness value of about 82 HV. In the present study, the hardness value is 30% higher than the value obtained by Kandemir et al. [33], and 10% higher than the result obtained by Lotfpour et al. [41].

**Figure 11:**
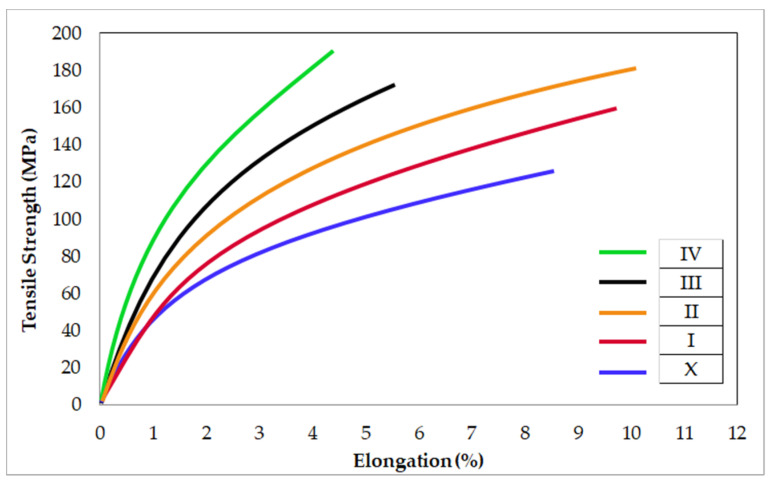
The tensile strength–elongation (%) curves of Mg2Zn-xAl_2_O_3_ composite specimens.

Figure 11 shows the tensile strength-elongation (%) curves of Mg2Zn-xAl_2_O_3_ composite specimens. It is seen that the tensile strength, yield strength, and elongation values of Al_2_O_3_ reinforced composites increased compared to Mg2Zn alloy. Depending on the increasing Al_2_O_3_ reinforcement ratio, the tensile strength increased steadily. Elongation (%) increases when Al_2_O_3_ reinforcement up to 4% by weight is added. However, elongation (%) decreases with the addition of more Al_2_O_3_ than this ratio. This result is in agreement with the investigation reported in literature [62,63]. The tensile strength of the Al_2_O_3_ reinforced specimens increases because of the addition of hard alumina particles in a softer matrix. In addition, fine-grained structures are thought to be effective in the development of tensile strength of specimens. It has also been stated in previous studies [31,47] that hard structures, which are densely located at the grain boundaries, act as barriers and make dislocation movements difficult. According to the literature [64], it may be also attributable to synergetic strengthening mechanisms including difference in elastic modulus strengthening, load-transfer strengthening, and difference in thermal expansion strengthening. Significant improvements in tensile strengths are observed in alumina particulate reinforced specimens in comparison composites Mg3Zn-5HA [17], AZ91-0.5 GNP [33], and Mg2Zn-5Cu [41]. The highest tensile strength value of approximately 191 MPa was from the specimen with 8 wt.% Al_2_O_3_. Considering the best corrosion results in the present study, the tensile strength of the specimen containing 2% alumina was approximately 160 MPa. This value is 26% higher than tensile strengths of Mg2Zn in present study, 11% higher than AZ91 [33], 19% higher than Mg2Zn-5Cu [41], and 33% higher than AZ91-2 wt.% SiC [17], respectively. A comparative presentation of the mechanical properties obtained in present study with some literature studies is given in Table 4.

## 4. Conclusions

In the present study, Al_2_O_3_ reinforced Mg2Zn matrix magnesium composites were successfully produced through the hot pressing method. The hardness and corrosion properties were investigated experimentally. The results of the analyses are as follows:

The risk of exposure of Mg powders to oxidation from the mixing process to the sintering stage has been eliminated due to the paraffin coating technique. MgMCs were produced with high relative density ratios by the hot pressing method.

The added Al_2_O_3_ particle reinforcements were homogeneously distributed at the grain boundaries and they were effective in grain refinement. Alumina reinforcement significantly improved the mechanical properties. Maximum hardness and tensile strength were obtained from the specimen containing 8 wt.% Al_2_O_3_ reinforcement. In the specimens where the alumina reinforcement ratio increased up to 4 wt.%, an increase in the % elongation values was achieved and the highest value was obtained from this specimen.

After immersion, Mg(OH)_2_ and the apatite structures were generally determined on the specimen surfaces. Apatite structures were densely formed on the surface of specimen (I) only. Apatite structures exhibited a permanent and protective behavior, resulting in a significant reduction in the corrosion rate and the amount of evolved H_2_ gas. Corrosion resistance is significantly reduced in specimens containing a high reinforcement ratio. As the corrosion resistance decreased, the amount of evolved H_2_ gas increased. When Al_2_O_3_ reinforcement is added at a low ratio (2 wt.%), galvanic corrosion is the minimum at the grain boundaries. This allows the formation of the apatite structure which improves the corrosion properties of the magnesium matrix composite material.

Paraffin coating technique enabled the production of other Mg-based alloys and composites by powder metallurgy method. By using the hot pressing method, there will be no need for an additional production process and this will help the development of the microstructure of different Mg-based materials. In applications where the mechanical and corrosion resistance of Mg based composites are required together, it is recommended to add 2 wt.% Al_2_O_3_ to the relevant composite content.

## Figures and Tables

**Table 1:** The chemical composition and nomenclature of the matrix alloy.

Matrix Code	Matrix Nomenclature	Mg (wt.%) Purity: 99.8% Size: <45 µm	Zn (wt.%) Purity: 99.9% Size: <10 µm
X	Mg2Zn	98	2

**Table 2:** The chemical compositions and nomenclatures of the Mg2Zn-xAl_2_O_3_ composite specimens.

Composite Code	Composite Nomenclature	Mg2Zn (wt.%)	Al_2_O_3_ (wt.%) Purity: 99.8% Size: <5 µm
X	Mg2Zn-0 wt.% Al_2_O_3_	100	0
I	Mg2Zn-2 wt.% Al_2_O_3_	98	2
II	Mg2Zn-4 wt.% Al_2_O_3_	96	4
III	Mg2Zn-6 wt.% Al_2_O_3_	94	6
IV	Mg2Zn-8 wt.% Al_2_O_3_	92	8

**Table 3:** A comparative presentation of the corrosion properties obtained in present study with some literature studies.

Material	Corrosion Rate (mm/Year)	Weight Loss (%)	Ref
Mg2Zn + 2 wt.% Al_2_O_3_	2.5	9	PS
AM100 + 25 vol.% Al_2_O_3_	7	-	[20]
ZC63 + 25 vol.% Al_2_O_3_	9.5	-	[20]
Mg3Zn + 5HA	-	27	[17]
Mg0.3GNP	4	-	[57]
Mg0.5Zr0.5GNP	18	-	[19]

PS: Present study. GNP: graphene nanoplatelet.

**Table 4:** A comparative presentation of the mechanical properties obtained in present study with some literature studies.

Material	Hardness (HV)	Tensile Strength (MPa)	Elongation (%)	Ref
Mg2Zn-2 wt.% Al_2_O_3_	72.2	160	9.8	PS
Mg2Zn-8 wt.% Al_2_O_3_	89.9	191	4.4	PS
AZ91-2 wt.% SiC	75	120	4	[62]
AZ91-8 wt.% SiC	85	145	3.5	[62]
Mg/Mg2Si-HO		145		[65]
Mg/Mg2Si-HR		155		[65]
Mg0.5Zr0.5GNP	53	-	-	[19]
Mg3Zn-0.5Cu	62	171	15	[41]
Mg3Zn-5Cu	80	135	6	[41]
AZ91-0.5 wt.% GNP	69	-	-	[33]

PS: Present study.

## Data Availability

All data generated or analyzed during this study are included in this article.
